# Humeral head size predicts baseplate lateralization in reverse shoulder arthroplasty: a comparative computer model study

**DOI:** 10.1016/j.jseint.2023.11.015

**Published:** 2023-12-14

**Authors:** Stefan Bauer, William G. Blakeney, Arnaud Meylan, Jaad Mahlouly, Allan W Wang, Arnaud Walch, Luca Tolosano

**Affiliations:** a Chirurgie de l’épaule et du coude, Service d'Orthopédie et de Traumatologie, Ensemble Hospitalier de la Côte, Morges, Switzerland; b School of Surgery, University of Western Australia, Perth, WA, Australia; c Department of Orthopaedic Surgery, Royal Perth Hospital, Perth, WA, Australia; d Centre Hospitalier Universitaire Vaudois, Lausanne, Switzerland; e CHU de Lyon, Groupement Hospitalier Edouard Herriot, Lyon, France

**Keywords:** Reverse shoulder arthroplasty, Neutral anatomic lateralization, Body height, Native humeral head size, Neck shaft angle, Glenosphere size, Notching, Distalization

## Abstract

**Background:**

In reverse shoulder arthroplasty (RSA), the ideal combination of baseplate lateralization (BL), glenosphere size (GS), and glenosphere overhang (GOH) with a commonly used 145° neck shaft angle (NSA) is unclear. This is the first study evaluating correlations of body height (BH), humeral head size (HS), glenoid height (GH), and association of gender with best glenoid configurations for range of motion (ROM) maintaining anatomic lateralization (aLAT) for optimized muscle length in 145° and less distalized 135° RSA.

**Methods:**

In this computer model study, 22 computed tomographies without joint narrowing were analyzed (11 male/female). A standardized semi-inlay 145° platform stem was combined with 20 glenoid configurations (baseplate [B] 25, 25 + 3/+6 lateralized [l], 29, 29 + 3/6l combined with glenosphere 36, 36 + 2 eccentric [e], 36 + 3l, 39, 39 + 3e, 39 + 3l , 42, 42 + 4e). Abduction-adduction, flexion-extension, external rotation-internal rotation, total ROM (TROM), and total notching relevant (TNR) ROM were computed, best TROM models respecting aLAT (-1 mm to +1 mm) and HS/GH recorded. Second, the 145° models (Ascend Flex stem; Stryker, Kalamazoo, MI, USA) were converted and compared to a 135° inlay RSA (New Perform stem; Stryker, Kalamazoo, MI, USA) maintaining GOH (6.5-7 mm) and aLAT.

**Results:**

Best 145° models had eccentric glenospheres (mean BL: 3.5 mm, GOH 8.8 mm, GS 38.1 mm, distalization 23 mm). The 135° models had concentric glenospheres, mean BL 3.8 mm, GOH 6.9 mm, GS 39.7 mm, and distalization 14.1 mm. HS showed the strongest positive correlation with BL in 145° and 135° models (0.65/0.79). Despite reduced GOH in smaller females with a 135° NSA, adduction, external rotation, extension, TNR ROM, and TROM were significantly increased (*P* = .02, *P* = .005, *P* = .005, *P* = .004, *P* = .003), abduction however reduced (*P* = .02). The same trends were seen for males.

**Conclusion:**

HS is a practical measure in surgery or preoperatively, and the strong positive correlation with BL is a useful planning aid. Despite reduction of GOH, conversion to a less distalized 135° NSAinlay design is powerful to maintain and even significantly increase all components of TNR ROM (extension/external rotation/adduction) in small females with the drawback of reduced abduction which may however be compensated by scapula motion. Lateralization with a less distalized 135° RSA optimizes muscle length, may facilitate subscapularis repair, and maintains highest rigid body motion.

Choosing the correct amount of baseplate lateralization (BL) and an appropriate glenosphere size (GS) in reverse shoulder arthroplasty (RSA) can be a difficult task during planning and surgery. To date, the ideal amount and combination of BL, GS, and glenosphere overhang (GOH) as well as the best neck shaft angle (NSA) are still unclear. A 145° NSA with 4 mm of BL and 2 mm of inferior glenosphere eccentricity has been shown to provide the middle ground for RSA in a computer model.^1^ However, most computer modeling studies have used deidentified computed tomography (CT) scans evaluating computed rigid body motion (RBM) without taking gender, height, shoulder size, and muscle tensioning into account.^1^^,^^18^^,^^20^^,^^22^^,^^32^^,^^33^

Page and colleagues published data from the Australian National Joint Replacement Registry analyzing GS and cumulative revision rates. They reported that large glenospheres for males and middle-sized glenospheres for females improve implant survival.^27^ However, their data were mostly based on RSA using Grammont’s design with a 155° NSA with limited BL and with a medialized center of rotation (COR) such as the Delta III (DePuy, Raynam, MA, USA), SMR (Lima Corp.), and Aequalis (Tornier, Bloomington, MN, USA) as the 3 most frequent RSA included in the Australian National Joint Replacement Registry. The Delta III and Aequalis can be classified as medial-glenoid medial-humerus (MGMH) prostheses. The SMR has increased in-built glenoid baseplate and glenosphere lateralization of 5-6 mm compared to the Aequalis and Delta 3.^34^ Recent developments have moved to more lateralized designs with lower NSA between 145° and 135° classified as medial-glenoid lateral-humerus (MGLH), lateral-glenoid medial humerus (LGMH), and mixed designs of MGLH and LGMH called bipolar lateralization. BL and GOH have shown to increase impingement-free range of motion (ROM),^2^ but it can be difficult for surgeons during surgery to judge how much the baseplate should be lateralized and which GS to use prior to implanting the stem.^5^^,^^34^ It has been recommended to lateralize the humerus close to its neutral, anatomic position to optimize muscle fiber length and tension, deltoid wrapping, and to avoid overstuffing.^34^ However, anatomic lateralization (aLAT) must be evaluated in the context of distalization (DIS) of the humerus, a variable which was most important for RSA stability with Grammont’s medialized design^21^ and which can be beneficial for ROM associated with GOH to reduce notching with a higher NSA of 145° to 155°.^2^ Excessive DIS may have drawbacks for muscle fiber length, nerve, and soft tissue tension.^21^^,^^25^

The first objective of this controlled RSA computer model study was to find the glenoid configuration (BL, GS, and eccentricity) for each patient allowing for the best total glenohumeral ROM (TROM), using a 145° humeral stem. The model respected aLAT of the humerus (-1 mm to +1 mm), as a simplified model for optimized muscle fiber length. These findings were correlated with body height (BH), gender, glenoid height (GH), and the native humeral head size (HS). The second part of the study focused on the conversion of these best models to a more anatomic, less distalized 135° RSA with a new implant maintaining aLAT and the comparison of ROM between the 145° and 135° RSA models.

## Methods

In this computer modelling study, we analyzed 22 CT scans which were previously carried out for clinical evaluation. Patient demographics are shown in Table I.

**Table I:** Demographic and anatomical data (height, head size, and glenoid height) of the 22 patients who gave consent for the use of their data.

	Females	Males	All
Age	63-83 ∅ 74.6 years	62-82 ∅ 71.1 years	62-83 ∅ 72.9 years
Height	151-172 ∅ 161 cm	170-190 ∅ 176 cm	151-190 ∅ 169 cm
Head size	41-48 ∅ 43.1 mm	46-52 ∅ 50.5 mm	41-52 ∅ 46.8 mm
Glenoid height	29-36 ∅ 32.5 mm	35-41 ∅ 38.0 mm	29-41 ∅ 35.3 mm

Approval of the ethics committee for retrospective analysis of the scans was obtained.

Patient identity was protected and blinded for the study analysis. Included patients had undergone CT scans for preoperative planning for RSA, with an etiology of massive rotator cuff tears without joint space narrowing. The inclusion criteria consisted of patients with Hamada grade 1-2 cuff tear arthropathies^17^ and Sirveaux E0-type glenoids.^28^ Degenerative medialization of the humerus was considered as an exclusion criterion.

Blueprint software (version 3.0.1; Imascap, Brest, France) was used to analyze the digital imaging and communications in medicine images of the CT scans.

**Figure 1:**
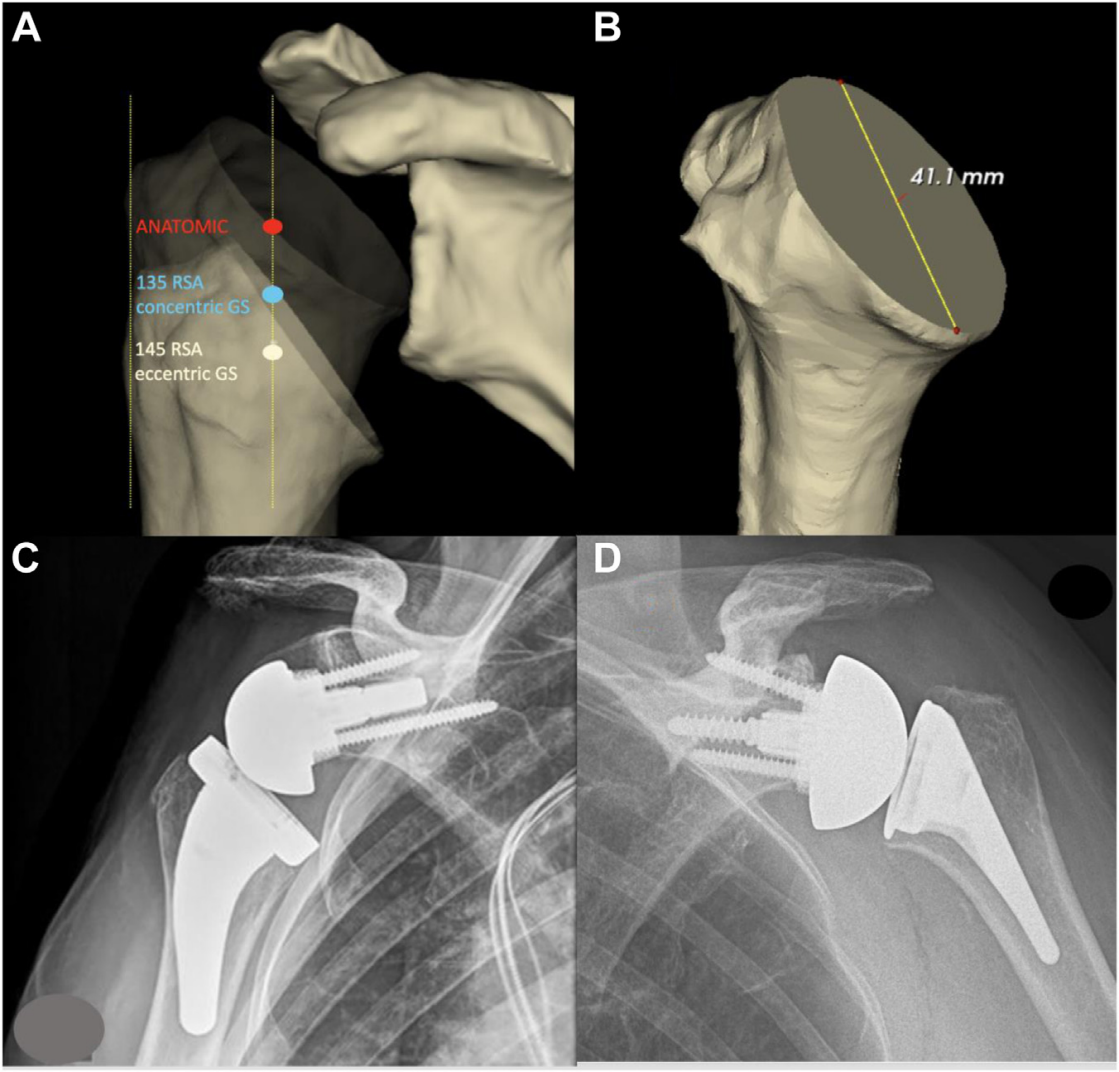
(**A**) Anatomic lateralization and differences of distalization in 145º (*white*), 135º (*blue*) models, and anatomic state (*red*). (**B)** Humeral head osteotomy diameter representing head size. (**C-D**) Example radiographs of 145º and 135º RSA corresponding to 145º (*white*) and 135º (*blue*) models in (**A**). *RSA*, reverse shoulder arthroplasty.

The software performed an automated segmentation prior to automatically calculating the glenoid version and inclination as previously described^31^ and prior to computing a neutral reference scapular plane based on automatic 3-dimensional (3D) reconstruction of all 3D points of the scapula body. Furthermore, the best-fit native humeral head osteotomy diameter was automatically calculated by a software algorithm which was manually corrected in a 3D model and in separate planes by 2 experienced shoulder surgeons until agreement on the sizing of the humeral head osteotomy diameter was reached. This humeral head measurement was taken at the anatomical neck of the humerus as an independent measure for glenohumeral size not related to the final tray position and osteotomy of the semi-inlay 145° platform RSA and it was used to represent the humeral HS (Fig. 1*B*) since it is expected not to be influenced by flattening of the humeral head in osteoarthritis, cuff tear arthropathy, and osteonecrosis and thought to be proportional to other humeral HS measurements and glenohumeral size.

As a control in these shoulders without joint space narrowing, the GH was measured in the inferior-superior axis of the mid-coronal plane computed by the software and by the same surgeons until agreement was reached.^13^

The automated measurement process, reference points, axis, and planes have previously been validated and published.^6^ For each patient’s CT scan, 20 different virtual glenoid configuration models were created with the software prior to simulating glenohumeral ROM computed as RBM limited by impingement detection between the scapula and the prosthesis for each model. Each model consisted of an Aequalis Ascend Flex stem and Perform Reversed baseplate shoulder arthroplasty system (Example radiograph in Fig. 1*C*, Stryker, Kalamazoo, MI, USA). The modular humeral implant was kept constant for all models and was virtually implanted with a 145° NSA which is commonly used in clinical practice.^1^ The virtual humeral osteotomy was performed in physiological retrotorsion and determined by the software algorithm without scanning the elbow. For a semi-inlay implant position, the highest point of the tray was aligned with the summit of the greater tuberosity and to achieve this, the osteotomy was lowered parallel to the level of the anatomical neck. The metaphyseal tray was chosen with a high eccentricity of 3.5 mm and dialed to position 6 medializing the humerus which is common practice for RSA with this lateralizing, flexed short stem. For all models, the thinnest humeral insert of +6 mm was chosen since the implant is clinically known to be “tight”. The glenoid implant was positioned according to a semi-automated, standardized algorithm as follows: Each baseplate was automatically positioned in 0° of inclination and 0° of version by the software according to a computed standardized plane as previously described.^2^ In this standardized plane, a 25 mm or 29 mm baseplate (Aequalis perform reversed; 25 mm for 36 mm and 39 mm glenospheres and 29 mm for a 42 mm glenosphere) was positioned flush at the level of the inferior extent of the glenoid, in neutral position of lateralization referring to the central post of the baseplate (0 mm as displayed by the software) without any further manual adjustments. Twenty different RSA model configurations were tested, being composed of 6 baseplate types (25 mm; 25 mm + 3 mm lateralization [l]; 25 mm + 6 mm lateralization; 29 mm; 29 + 3 mm lateralization; 29 + 6 mm lateralization) and 8 different glenospheres (36 mm; 36 mm + 2 mm eccentricity [e]; 39 mm; 39 mm + 3 mm eccentricity; 42 mm; 42 mm + 4 mm eccentricity; 36 mm + 3 mm lateralization [l] and 39 mm + 3 mm lateralization) without combining 25 mm baseplates with a 42 mm glenosphere and 29 mm baseplates with a 36 mm or 39 mm glenosphere. The following glenoid configurations were tested: (1) 25/36, (2) 25/36 + 2e, (3) 25/39, (4) 25/39 + 3e, (5) 29/42, (6) 29/42 + 4e, (7) 25 + 3l/36, (8) 25 + 3l/36 + 2e, (9) 25 + 3l/39, (10) 25 + 3l/39 + 3e, (11) 29 + 3l/42, (12) 29 + 3l/42 + 4e, (13) 25 + 6l/36, (14) 25 + 6l/36 + 2e, (15) 25 + 6l/39, (16) 25 + 6l/39 + 3e, (17) 29 + 6l/42, (18) 29 + 6l/42 + 4e, (19) 25 + 3l/36 + 3l, and (20) 25 + 3l/39 + 3l. Medial-lateral change of the humerus (Lat) and DIS were computed by the software, displayed in 1 mm increments, comparing the preoperative anatomic position to the humeral position after RSA implantation as shown in Fig. 1*A*. All RSA models were tested for impingement-free ROM in 3 planes computed by the software: Abduction (AB) to adduction (AD), external rotation (ER) to internal rotation (IR), flexion (FL) to extension (EX), and at 0° of abduction. Total notching relevant ROM (TNR ROM) was defined as the sum of ER, EX, and AD and total ROM (TROM) as the sum of all ROM values.

In the second part of the study, the parameter DIS was examined further and reduced by 2 steps: First, by converting the constant 145° semi-inlay humeral implant to a 135° inlay stem (Fig. 1*A* and example radiograph in Fig. 1*D*: Perform, Stryker, Wright medical, Bloomington, MN, USA). For this implant, a virtual anatomical osteotomy was used as calculated by the software without lowering the level of humeral resection for this inlay stem. aLAT was maintained, if possible, with a +0 mm or +3 mm insert. In 2 male patients, an increase in BL was necessary by +3 mm. Second, we converted all eccentric glenospheres to concentric glenospheres maintaining at least 6.5 mm overhang: 36 + 2e to 39, 39 + 3e to 39 (25 mm baseplate) and 42 + 4e to 42 (29 mm baseplate). Fig. 1*A* shows the decrease of DIS by these steps and Fig. 1*D* a clinical example radiograph with a concentric 39 G and the 135° inlay stem in comparison to the 145° platform semi-inlay implantation in Fig. 1*C*.

### Outcome parameters and variables

TROM was the primary outcome variable within the confines of aLAT (-1 mm to +1 mm) for the first part of the study with the 145° platform semi-inlay RSA. Neutral aLAT was considered a prerequisite to select the best glenohumeral TROM model for each patient. Secondary variables were gender, BH, HS, GH, BL, GS, GOH, and TNR ROM for each selected best TROM model respecting neutral aLAT.

In the second part of the study, after conversion of best models to a constant 135° RSA with concentric 39 and 42 glenospheres, the parameters DIS as well as TROM, TNR ROM, AB-AD, ER-IR, and FL-EX were compared between best 145° models and 135° models after the aforementioned 2-step reduction of DIS.

### Statistical analysis

**Figure 2:**
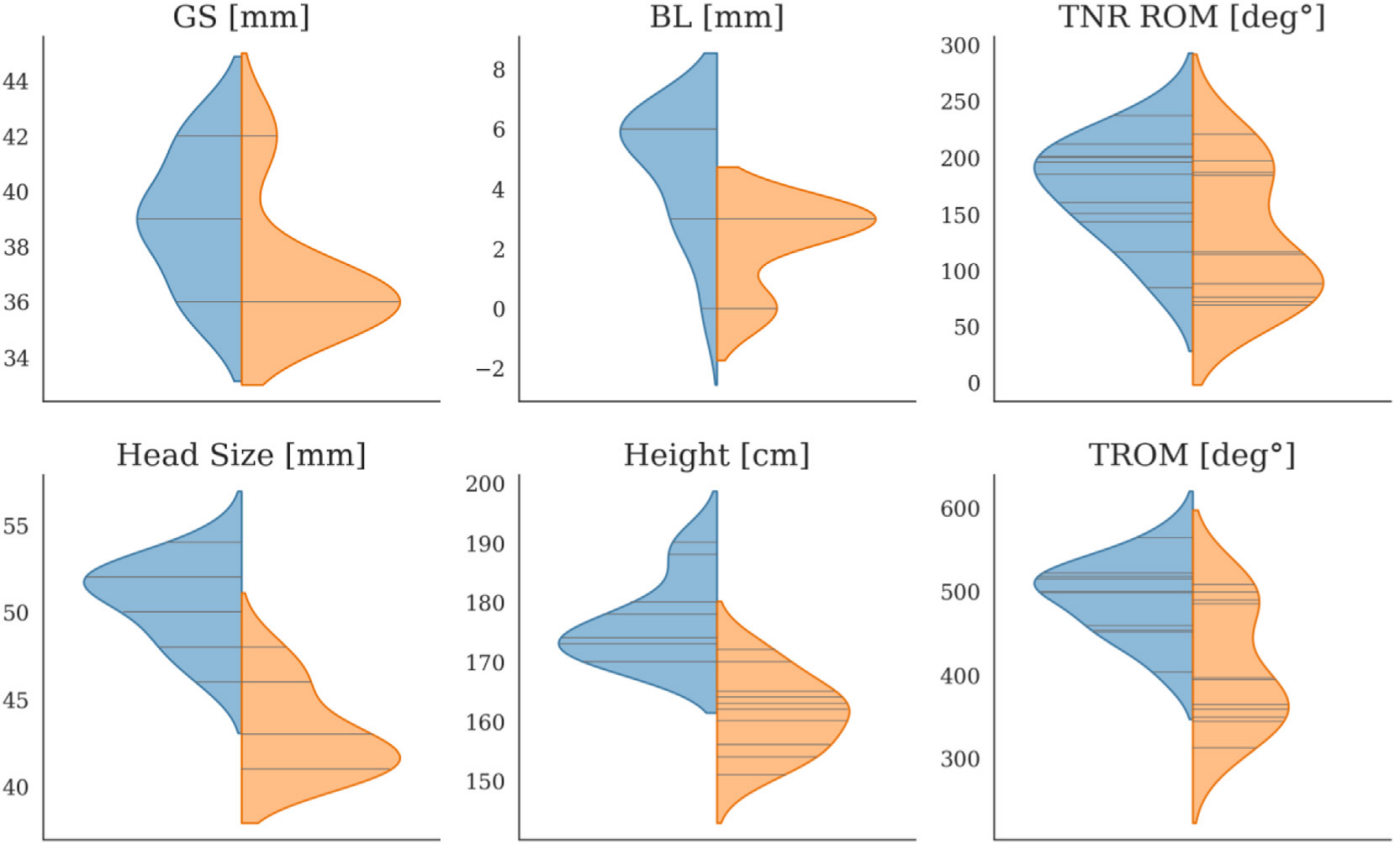
Violin plots showing the distribution of glenosphere size (GS), baseplate lateralization (BL), Head Size (HS), Height, TNR ROM, and TROM for male (*blue*) and female patients (*orange*). *TNR*, total notching relevant range of motion; *TROM*, total range of motion.

Descriptive statistics were used to select the best 145° RSA model as the highest TROM within the confines of aLAT (-1 mm to +1 mm) for each patient. The best glenoid configurations were illustrated by violin plots (Fig. 2) describing distributions for BL, GS, gender, BH, HS, TROM, and TNR ROM. DIS and ROM of the 145° and 135° models were displayed by descriptive statistics for the complete cohort, female and male patients. The ROM of models with a constant 145° platform semi-inlay design associated with eccentric glenospheres was compared with the ROM of models with a constant 135° inlay stem associated with concentric glenospheres using student’s *t*-tests for parametric data.

Pearson’s correlation statistics were computed to analyze the correlations between TROM, TNR ROM, height and HS, GH with BL, GOH and GS as well as the correlations between height, and HS and GH. A correlation coefficient of 0.8-1.0 was classified to be a very strong, 0.6-0.79 a strong, 0.4-0.59 a moderate, 0.2-0.39 a weak, and 0.0-0.19 a very weak positive correlation.

For all statistical analyses and illustrations, Matplotlib and Seaborn were used as Python data visualization libraries alongside with SciPy as Python scientific computing library for computing of correlations and *P* values which were considered significant for *P* < .05.

## Results

### Best models of 145° platform semi-inlay RSA

Primary and secondary outcome variables such as gender, height, HS, GS, BL, GOH, the breakdown of specific and combined ROM parameters, and LAT are shown in Tables I and II. All best 145° models had an eccentric glenosphere and all but 4 of 22 best models (82%) had a lateralized baseplate. The 4 best models without BL had a mean native humeral HS of 44 mm (range 41 mm-46 mm). Best models with a humeral HS of less than 47 mm (n = 11, mean 43.3 mm) had a mean GS of 36.5 mm, a mean BL of 1.9 mm, and the most frequent combination was 25 + 3/36 + 2e. Best models with a humeral HS of more than 47 mm (n = 11, mean 50.7 mm) had a mean GS of 39.5 mm, a mean BL of 4.9 mm, and the most frequent combination was 25 + 6/39 + 3e.

**Table II:** Comparison of best 145° and less distalized 135° RSA models for LAT, BL, GS, GOH, ROM parameters, and distalization.

Gender	Females	Males	Overall
Lateralization (LAT)			
145° RSA∗	−1 to (+1) $\bar{\text{x}}$ 0.1 mm	−1 to (+1) $\bar{\text{x}}$ 0.4 mm	−1 to (+1) $\bar{\text{x}}$ 0.2 mm
135° RSA†	−1 to (+3) $\bar{\text{x}}$ 0.9 mm	−1 to (+1) $\bar{\text{x}}$ 0.2 mm	−1 to (+2) $\bar{\text{x}}$ 0.4 mm
Baseplate Lateralization (BL)			
145° RSA∗	0 to (+3) $\bar{\text{x}}$ 2.2 mm	0 to (+6) $\bar{\text{x}}$ 4.9 mm	0 to (+6) $\bar{\text{x}}$ 3.5 mm
135° RSA†	0 to (+3) $\bar{\text{x}}$ 2.2 mm	3 to (+6) $\bar{\text{x}}$ 5.2 mm	0 to (+6) $\bar{\text{x}}$ 3.8 mm
Glenossphere Size (GS)			
145° RSA∗	36-42 $\bar{\text{x}}$ 37.1	36-42 $\bar{\text{x}}$ 39.0	36-42 $\bar{\text{x}}$ 38.1
135° RSA†	39-42 $\bar{\text{x}}$ 39.5	39-42 $\bar{\text{x}}$ 39.8	39-42 $\bar{\text{x}}$ 39.7
Glenosphere Overhang (GOH)			
145° RSA∗	7.5-10.5 $\bar{\text{x}}$ 8.05 mm	7.5-10.5 $\bar{\text{x}}$ 9.45 mm	7.5-10.5 $\bar{\text{x}}$ 8.75 mm
135° RSA†	6.5-7.0 $\bar{\text{x}}$ 6.91 mm	6.5-7.0 $\bar{\text{x}}$ 6.86 mm	6.5-7.0 $\bar{\text{x}}$ 6.89 mm
TNR ROM 145° RSA∗	69-221 $\bar{\text{x}}$ 128°	84-237 $\bar{\text{x}}$ 173°	69-237 $\bar{\text{x}}$ 150°
TNR ROM 135° RSA†	114-228 $\bar{\text{x}}$ 198°	111-241 $\bar{\text{x}}$ 184°	111-241 $\bar{\text{x}}$ 191°
	*P* = .004∗	*P* = .61	*P* = .01∗
TROM 145° RSA∗	312-508 $\bar{\text{x}}$ 409°	403-564 $\bar{\text{x}}$ 491°	312-564 $\bar{\text{x}}$ 450°
TROM 135° RSA†	417-527 $\bar{\text{x}}$ 494°	435-545 $\bar{\text{x}}$ 501°	417-545 $\bar{\text{x}}$ 497°
	*P* = .003∗	*P* = .62	*P* = .01∗
Abduction (AB) to Adduction (AD)			
145° RSA∗	AB (61-91) to AD (17-39) $\bar{\text{x}}$ AB 77° to AD 27°	AB (71-105) to AD (17-60) $\bar{\text{x}}$ AB 87° - AD 42°	AB (61-105) to AD (17-60) $\bar{\text{x}}$ AB 82° to AD 35°
135° RSA†	AB (51-82) to AD (27-46) $\bar{\text{x}}$ AB 66° to AD 36°	AB (54-95) to AD (32-64) $\bar{\text{x}}$ AB 77° to AD 47°	AB (51-95) to AD (27-64) $\bar{\text{x}}$ AB 72° to AD 42°
	AB *P* = .02∗/AD *P* = .02∗	AB *P* = .11/AD *P* = .25	AB *P* = .01∗/AD *P* = .04∗
External (ER) to Internal Rotation (IR)			
145° RSA∗	ER (32-62) to IR (80-106) $\bar{\text{x}}$ ER 45° to IR 94°	ER (38-74) to IR (71-114) $\bar{\text{x}}$ ER 58° to IR 100°	ER (32-74) to IR (71-114) $\bar{\text{x}}$ ER 51° to IR 97°
135° RSA†	ER (46-70) to IR (80-104) $\bar{\text{x}}$ ER 57° to IR 94°	ER (40-82) to IR (73-108) $\bar{\text{x}}$ ER 57° to IR 94°	ER (40-82) to IR (73-108) $\bar{\text{x}}$ ER 57° to IR 94°
	ER *P* = .005∗/IR *P* = .93	ER *P* = .91/IR *P* = .27	ER *P* = .12/IR *P* = .32
Flexion (FL) to Extension (EX)			
145° RSA∗	FL (91-128) to EX (20-120) $\bar{\text{x}}$ FL 110° to EX 56°	FL (102-151) to EX (32-120) $\bar{\text{x}}$ FL 133° to EX 72°	FL (91-151) to EX (20-120) $\bar{\text{x}}$ FL 121° to EX 64°
135° RSA†	FL (88-139) to EX (41-120) $\bar{\text{x}}$ FL 109° to EX 104°	FL (97-151) to EX (39-120) $\bar{\text{x}}$ FL 128° to EX 79°	FL (88-151) to EX (39-120) $\bar{\text{x}}$ FL 119° to EX 92°
	FL *P* = .96/EX *P* = .005∗	FL *P* = .55/EX *P* = .63	FL *P* = .67/EX *P* = .01∗
Distalization (DIS) 145° RSA∗	17.5-27.1 $\bar{\text{x}}$ 22.8 mm	19.4-26.9 $\bar{\text{x}}$ 23.2 mm	17.5-27.1 $\bar{\text{x}}$ 23.0 mm
Distalization (DIS) 135° RSA∗	10.5-22.1 $\bar{\text{x}}$ 15.8 mm	13.4-22.9 $\bar{\text{x}}$ 16.8 mm	11.8-23.9 $\bar{\text{x}}$ 16.3 mm
Distalization (DIS) 135° RSA†	9.5-17.2 $\bar{\text{x}}$ 14.0 mm	10.8-19.1 $\bar{\text{x}}$ 14.2 mm	8.5-19.9 $\bar{\text{x}}$ 14.1 mm

*TNR ROM*, total notching relevant range of motion; *TROM*, total range of motion; *RSA*, reverse shoulder arthroplasty; *ROM*, range of motion.

$\bar{\text{x}}$ indicates average value.

∗ Eccentric 36, 39, and 42 glenospheres.

† concentric 39 and 42 glenospheres. Significance: *P* < .05∗.

The distribution of number of best 145° models for 6 variables is illustrated in Fig. 2 by violin plots with sticks and further specified in Table II.

The most frequent GS was 39 + 3e for males and 36 + 2e for females, the most frequent BL was 6 mm for males and 3 mm for females. Gender was associated with the amount of BL and the GS as shown in the violin plots in Fig. 2. The mean height for males was 176 cm and for females 161 cm, HS 50.5 mm and 43.1 mm, respectively, mean TNR ROM and TROM 171° and 491°, respectively, for males and 128° and 409°, respectively, for females indicating a larger geometric space available for impingement-free ROM beyond physiological and clinical requirements in males and associated with increased GOH and GS in males as shown in Table II. Correlations of ROM and anatomic parameters with 145° and 135° RSA models are shown in Tables III and IV.

**Table III:** Correlations of anatomic parameters, best 145° RSA∗ models and ROM.

	Baseplate lateralization (BL)	Glenosphereoverhang (GOH)	Glenosphere size (GS)	Body height	Head size	Glenoid height
TROM						
145° RSA∗	0.75	0.69	0.65	0.59	0.71	0.64
TNR ROM						
145° RSA∗	0.63	0.70	0.69	0.37	0.53	0.45
Body Height	0.55	0.41	0.32	1.00	0.84	0.72
Head Size	0.65	0.61	0.50	0.84	1.00	0.92
Glenoid Height	0.62	0.50	0.42	0.72	0.92	1.00

*TROM*, total range of motion; *TNR ROM*, total notching relevant range of motion; *RSA*, reverse shoulder arthroplasty; *ROM*, range of motion.

∗ Eccentric 36, 39, and 42 glenospheres.

**Table IV:** Correlations of anatomic parameters and 135° models.

	Baseplate lateralization (BL) 135° RSA∗	Glenosphere overhang (GOH) 135° RSA∗	Glenosphere size (GS) 135° RSA∗
Body Height	0.63	−0.11	0.11
Head Size	0.79	−0.22	0.22
Glenoid Height	0.74	−0.19	0.19

∗ Concentric 39 and 42 glenospheres.

TROM showed the strongest positive correlation with BL (0.75) and TNR ROM showed the strongest positive correlation with GOH (0.70) for the best 145° RSA models. Height showed a moderate positive correlation with BL (0.55) for 145° models and a strong positive correlation for 135° models (0.63). GH showed a strong positive correlation with BL for 145° and 135° models (0.62 and 0.74). HS showed a very strong positive correlation with GH (0.92) and BH (0.84), and strongest positive correlation with BL for 145° and 135° models (0.65 and 0.79) and a moderate positive correlation with GS for 145° models (0.5). HS, as 1 of 3 patient-specific preoperative variables, showed the strongest positive correlation with implant configuration variables.

### Distalization (DIS)

As shown in Table II, for the 145° semi-inlay platform stem, the DIS averaged 23 mm (17.5 to 27.1 mm) and was reduced with a constant 135° inlay stem maintaining eccentric glenospheres by a mean of 6.7 mm in all patients. It was further reduced by a mean of 2.2 mm by the use of concentric glenospheres (medium to large size: 39 and 42 mm) to a mean total DIS of 14.1 mm (8.5 to 19.9 mm).

### Range of motion (ROM), glenosphere overhang (GOH), and glenosphere size (GS)

The mean TROM for the 145° platform design with eccentric glenospheres was 450° (312° to 564°) with a large difference between females (mean 409°, 312° to 508°) and males (491°, 403° to 564°) as shown in Table II. The mean TNR ROM of the 145° platform design with eccentric glenospheres was 150° (69° to 237°), again with a large difference between females (mean 128°, 69° to 221°) and males (mean 173°, 84° to 237°). For 145° models, the mean GOH/GS was 8.05/37.1 mm for females and 9.45/39.0 mm for males with an overall mean GOH/GS of 8.75/38.1 mm.

After converting the constant 145° semi-inlay platform stem models with eccentric glenospheres to models with a constant 135° inlay stem with concentric glenospheres, reducing DIS while maintaining a GOH between 6.5 mm and 7 mm (females: mean 6.91 mm; males mean: 6.86 mm), the mean TROM as well as mean TNR ROM were increased for males (*P* = .62 and .61), significantly for females (*P* = .003 and .004) and overall (*P* = .01 for TROM and TNR ROM) and the large difference between females and males was not seen any more, associated with equalized GOH and approximation of GSs. For 135° models, the mean GOH/GS was 6.91/39.5 mm for females and 6.86/39.8 mm for males with an overall mean GOH/GS of 6.89/39.7 mm.

Analysis of specific ROM in the 3 planes examined showed that despite the small reduction in GOH, the conversion to a 135° inlay stem significantly improved ER and EX for females (*P* = .005 for both) and overall (*P* = .12 and .001) and the lowest values in females and males were elevated (ER: 32° to 46°; EX: 20° to 41°). In the 135° models as shown in Table II, IR remained stable overall, slightly reduced for males associated with less GOH in their models and stable for females. FL remained stable comparing the 145° with the 135° models, AD increased nonsignificantly for males (*P* = .25) and significantly for females (*P* = .02) and overall (*P* = .04). The only significant reduction of a specific ROM in the 135° models was seen for abduction in females (mean 11°, *P* = .02) and overall (mean 10°, *P* = .01). This was nonsignificant for males (mean 10°, *P* = .11).

## Discussion

This computer modeling study looked at best ROM for RSA models in male and female patients, respecting aLAT. The key finding is that of all patient-specific parameters, the native humeral HS showed the strongest positive correlation with the amount of BL to optimize ROM for both 135° and 145° models. The frequently used 145° semi-inlay platform design had been shown to be the middle ground for optimized ROM with associated BL and glenosphere eccentricity.^1^ These positive correlations are stronger than the positive correlations of BH and GH.

Gender was also associated with the amount of BL for 145° models. For humeral HSs of less than 47 mm, the most frequent configuration was 25 + 3/36 + 2e (7.5 mm GOH) which were converted to 25 + 3/39 (7 mm GOH) with the 135° design. In humeral HSs of more than 47 mm, the most frequent combination was 25 + 6/39 + 3e (10 mm GOH) which were converted to 25 + 6/39. The mean BL of all 145° ROM models was 3.6 mm and the mean glenosphere eccentricity 2.7 mm, close to Arenas-Miquelez findings for optimized ROM with a 145° NSA.^1^ The mean native HS in our study (46.8 mm; females: 43.1 mm; males 50.5 mm) was representative and in keeping with Boileau’s and Walch’s benchmark study of the spectrum and anatomy of the proximal humerus which found a mean native HS diameter of 46.2 mm.^9^ The native humeral HS measured as the anatomical humeral osteotomy diameter is a very useful predictor for the geometrical space available and since less vulnerable to degenerative changes compared to the glenoid, it can help surgeons with the difficult task of planning adequate BL prior to fine-tuning soft tissue tension and stability during surgery.

The second very important finding is the potential of a 135° NSA to significantly increase ROM relevant for notching, such as ER, EX, and AD in smaller female patients despite reduction of GOH. The comparison of specific ROM of the distalized 145° semi-inlay RSA with the models after conversion to a 135° NSA shows the huge potential of a 135° NSA. EX, ER, and AD were significantly increased for smaller females elevating all lower values of EX, ER, and AD. FL and IR were maintained and the only drawback was reduced ABD brought about by both, a lower NSA and less DIS which may clinically be compensated by scapula motion. The large differences of TROM and TNR ROM between females and males in the 145° models were mainly brought about by the differences in GOH and GS (Table II) associated with the higher NSA. After conversion of the 145° models to a 135° NSA with equalization of GOH and increasing GS in females, these differences were not seen any more.

Other findings in 145° models are that TROM showed the strongest correlation with BL as previously reported^1^ and the second strongest with GOH. TNR ROM showed the strongest positive correlation with GOH and second strongest with BL. This is in keeping with previous findings that the combination of BL and GOH provides improved ROM with the potential to prevent notching.^1^^,^^2^

Why is there a trend toward neutral aLAT in RSA with a preference of lateralization on the glenoid side (LGMH) and why was progressive glenoid lateralization investigated in the computer models of this study? Grammont’s reverse shoulder design was characterized by medialization of the glenosphere, COR, and humerus (MGMH) with a 155° NSA.^11^^,^^19^ This development came about after early failure of more anatomically lateralized constrained prosthesis, which failed at the glenoid implant interface due to overlateralization, constrained design, and rudimentary fixation technology.^12^ Grammont’s principles included an increase of the biomechanical lever arm of the deltoid by medialization of the COR, re-tensioning of deltoid fibers by DIS, since lateralization had to be reduced to secure glenoid fixation, and the use of a semi-constrained design to provide stability. It therefore provided better mid-term and long-term outcomes than the previous designs eliminating failure of glenoid fixation.^10^^,^^29^ On the other hand, Grammont’s RSA went along with multiple drawbacks such as a loose remaining rotator cuff with weakness in external rotation, reduced external and internal rotation due to glenosphere medialization associated with a high incidence of notching, instability in abducted positions when the deltoid was de-tensioned, and loss of shoulder contour with excessive arm lengthening.^5^^,^^34^ Frankle was the first to address these by lateralization of the COR away from the glenoid bone interface with a nonhemispherical glenosphere, less DIS, and a 135° NSA.^15^ Boileau followed this development with bony BL improving rotation, shoulder contour, decreasing notching, and instability, however maintaining DIS at first due to the 155° and 145° NSA.^7^^,^^8^ Reduced complication rates and benefits were confirmed in a systematic review and meta-analysis.^14^^,^^26^ Most recently, metal augmentation has been introduced to lateralize the baseplate with emerging high-level evidence of equivalent outcomes compared to bony BL.^30^

Levin et al have published the results of Frankle’s and DiGiacomo’s research group on shoulder arthroplasty design, shoulder size, moment arms, and muscle fiber length.^24^ They compared muscle fiber length of LGMH and MGLH RSA lateralization.^4^^,^^24^ They found that although biomechanically, the deltoid moment arm and torque were increased for MGLH,^3^ the muscle fiber length of the MGLH design was located on the descending portion of the Blix curve^16^ which may compromise deltoid force generation, whereas the LGMH design remained close to the anatomic operating range of deltoid muscle fibers. The important difference between Frankle’s “anatomical” LGMH design^15^^,^^24^ and the LGMH RSA of our 145° models are the NSA of 135° and the amount of DIS/GOH which averaged 23 mm/8.8 mm in our study. This amount of DIS of the humerus may overlengthen the cuff and deltoid fibers and may reach critical safety limits of nerve distension as previously published.^23^^,^^25^ Biomechanically, the moment arm of the deltoid and cuff is reduced if the COR is lateralized toward the native COR of the humerus and increased by medialization and DIS moving the deltoid and cuff insertions away from the COR. It is largest for RSA with MGLH and considered “inbetween” for LGMH RSA. However, the more anatomical LGMH design shifts the deltoid and cuff muscle length to the plateau of the Blix curve optimizing muscle contractility.^24^ For the second part of our study, we have therefore converted the constant 145° platform stem decreasing overall DIS. First, by decreasing the NSA to 135° with a true inlay design and second, using concentric glenospheres while maintaining the lateralization of the COR (LGMH) and a constant GOH (6.5-7 mm).

There has been some controversy in the literature about the importance of GS in RSA. Page and coworkers have found registry evidence that larger glenospheres reduce cumulative revision rates.^27^ These data however seem to be influenced by a larger number of MGMH Grammont RSA being included in this registry, a design which benefits from larger glenospheres to achieve more lateralization, reduced notching, and improved stability. Werner and coworkers have reported on improved ROM with a larger 39 mm glenosphere compared to a 36 mm glenosphere, both modeled with a 29 mm baseplate in a computer study.^33^ A larger glenosphere was advantageous for ROM; however, this generalized conclusion seems to have been influenced by a small GOH of 3.5 mm with reduced ROM created by the combination of a 36 mm glenosphere with a 29 mm baseplate. In contrast to these findings, Lädermann showed improved ROM for a smaller eccentric 36 mm glenosphere and limitation of rotation in abduction of a larger 42 mm glenosphere.^20^ Use of a larger glenosphere in smaller patients may also compromise internal rotation since it may lead to overtensioning of the posterior capsule,^23^ or coracoid impingement. In our study, a larger glenosphere was exclusively selected within the confines of neutral aLAT and best glenohumeral ROM respecting the glenohumeral size and geometrical space available which may prevent the aforementioned detrimental effects described in Lädermann’s and Langohr’s studies. In our 145° study, the available geometric space for best ROM was most efficiently used for females and HS < 47 mm in most cases with +3 BL + 36 + 2e glenospheres and or for males and HS > 47 mm with +6 BL + 39 + 3e. Conversion to a 135° inlay design with less DIS on the humeral side allowed the use of concentric 39 mm glenospheres for most females and males with an average BH of 169 cm and HS of 46.8 mm with some outliers for large patients where we used a concentric 42 mm G. In contrast, in clinical practice, Frankle positions small nonhemispherical lateralized glenosheres centrally on the glenoid for his “anatomic” 135° RSA, 32 mm with +10 mm or +6 mm inbuilt lateralization for most males and females, respectively. It remains to be said that the ideal GS seems to depend on multiple parameters: (1) The height and positioning on the glenoid. (2) The overall design (LGMH, MGMH, MGLH). (3) The desired DIS since a larger concentric glenosphere can reduce DIS while maintaining overhang compared to a smaller eccentric glenosphere. (4) Stability, since a larger glenosphere can increase stability by increasing tension through lateralization and DIS.

Compared to previous RSA computer modeling in the literature, this study has a number of strengths. These consist of a controlled humeral design (145° + 6 insert and 135° + 0 to +3 insert) as a constant which was combined in the first part of the study with a large variety of controlled glenoid computer models with increasing BL, GOH, and size. These models were standardized and guided by neutral implant position according to the plane of the scapula computed by the software. A further strong point of this study is the balanced distribution of gender and size of included glenohumeral joints and their integrity without degenerative wear.

Limitations of this study are those associated with computer modeling of the glenohumeral ROM of RSA. First, scapulothoracic contribution to the shoulder ROM was not taken into account, which may compensate largely for reduced flexion and abduction (which was reduced in our 135° models) and to a lesser degree for restricted ROM with the arm at side. Second, soft tissue constraints and active muscle force generation cannot be accounted for which may lead to reduced active ROM. Third, in clinical practice, soft tissue tightness and small glenohumeral size may prevent BL as well as inferior GOH and its associated DIS. Load-sensor studies may improve our understanding of optimized joint reaction forces in different arm positions in the future. Fourth, there are further options to improve the glenohumeral ROM of RSA such as dialing the glenosphere eccentricity anteriorly or posteriorly which have not been investigated in this study. Fifth, the absolute implant measurements of this study are applicable for the frequently used RSA of this study but cannot be translated to a different geometry of other implants; however, the humeral HS remains an independent reference. Fifth and finally, neutral aLAT of the humerus in nonarthritic shoulders computed by the software as in this study can only be an approximation of anatomic muscle fiber length since lateralization was compared to the native humerus in a static neutral arm position. The analysis of the musculotendinous length relationships and humeral position change in different arm positions was not possible with the software used but has been studied by Levin et al^24^ in lateralized 135° models with reduced DIS. They have found that the LGMH design also investigated in our study was closest to anatomic musculotendinous length in different arm positions. Quantification of prosthetic humeral lateralization compared to premorbid state is currently not available due to degenerative glenoid wear and humeral deformity. However, with further development of statistical shape models, the premorbid anatomy of the humerus and glenoid will become available allowing measurements of muscle fiber length of the premorbid, arthritic, and reversed prosthetic state.

## Conclusion

In clinical practice, it is useful for surgical planning to have a preoperative estimate of the amount of lateralization required in RSA. Patient-specific HS showed the strongest positive correlation with adequate BL in this study. The NSA of 135° is a powerful parameter to maintain or increase all components of notching-relevant ROM (extension, external rotation, adduction) in small females. Lateralization with a less distalized 135° RSA go hand in hand as a combination to optimize muscle fiber length and ROM. They may facilitate subscapularis repair and maintain the highest passive RBM.

## Acknowledgments

Many thanks to the Australian Government for the support of this work as part of a Research Training Program PhD scholarship kindly granted for the main author (S.B.).

## Disclaimers:

Funding: No funding was disclosed by the authors.

Conflicts of interest: Stefan Bauer is a consultant for Stryker Osteonics SA without any personal payments; the Australian Government supported Dr. Bauer as part of a Research Training Program PhD scholarship kindly that is not related to the funding of this study. The other authors, their immediate families, and any research foundation with which they are affiliated have not received any financial payments or other benefits from any commercial entity related to the subject of this article.
